# A Novel Relapsing Fever Group *Borrelia* Isolated from *Ornithodoros* Ticks of the Brazilian Caatinga

**DOI:** 10.3390/microorganisms11020370

**Published:** 2023-02-01

**Authors:** Glauber M. B. de Oliveira, Sebastián Muñoz-Leal, Adriana Santodomingo, Barbara C. Weck, Álvaro A. Faccini-Martínez, Maurício C. Horta, Marcelo B. Labruna

**Affiliations:** 1 Departamento de Medicina Veterinária Preventiva e Saúde Animal, Faculdade de Medicina Veterinária e Zootecnia, Universidade de São Paulo, São Paulo 05508-270, SP, Brazil; 2 Department of Animal Science, Faculty of Veterinary Sciences, University of Concepción, Chillán 3800708, Chile; 3 Research Institute, Fundación Universitaria de Ciencias de la Salud, Bogotá 110110, Colombia; 4 Servicios y Asesorías em Infectología, Bogotá 110110, Colombia; 5 Servicio de Infectología, Hospital Militar Central, Bogotá 110110, Colombia; 6 Laboratório de Doenças Parasitárias, Universidade Federal do Vale do São Francisco, Petrolina 48903-435, PE, Brazil

**Keywords:** spirochetes, argasidae, isolation, dark-field microscopy, pernambuco

## Abstract

Tick-borne relapsing fever group (RFG) borreliosis remains neglected as a human disease and little is known on its maintenance in ticks and vertebrates, especially in South America. Therefore, this study investigated borrelial infection in *Ornithodoros* ticks collected in rodent-inhabited rock formations in the Brazilian semiarid region, within the Caatinga biome. Collected ticks (*Ornithodoros rietcorreai* and *Ornithodoros* cf. *tabajara*) were allowed to feed under laboratory conditions on guinea pigs, which had blood samples examined daily by dark-field microscopy. No spirochetes were visualized in the blood of any of four *O. rietcorreai-*infested guinea pigs. Contrastingly, spirochetes were visualized between 9 and 39 days after tick feeding in the blood of three guinea pigs, each infested with *O.* cf. *tabajara* ticks from a different locality. Guinea pig infection was confirmed by passages into experimental animals and by generating DNA sequences of *Borrelia* spp. from the blood of spirochetemic guinea pigs. Three *O.* cf. *tabajara* populations were infected by the same borrelial organism, which was characterized as a novel RFG agent (named as ‘*Candidatus* Borrelia caatinga’) based on 10 *Borrelia* loci (*rrs*, *flaB*, *glpQ*, *gyrB*, *clpX*, *pepX*, *pyrG*, *recG*, *rplB* and *uvrA*). We demonstrated that *O.* cf. *tabajara* is a competent vector of the novel *Borrelia* sp. isolates, although none of the infected rodents developed clinical illness.

## 1. Introduction

The spirochete genus *Borrelia* constitute bacteria that infect vertebrates, to whom they are transmitted by hematophagous vectors. With the exception of *Borrelia recurrentis,* transmitted by the human clothing louse (*Pediculus humanus humanus*), all *Borrelia* species are primarily transmitted by ticks [1,2]. Species of the genus *Borrelia* are known to infect a variety of vertebrate hosts (mammals, birds, reptiles) and have been divided into three main groups: (i) the Lyme group (LG), represented by *Borrelia burgdorferi* sensu lato genospecies that are associated with hard ticks (Ixodidae family); (ii) the Reptile-Echidna group (REPG), represented by organisms associated with hard ticks; and (iii) the Relapsing Fever group (RFG), which contains numerous organisms mainly associated with soft ticks (Argasidae family) and a few ones associated with hard ticks, in addition to the louse-borne *B. recurrentis* [1,2]. Although there has been a recent proposal to split borrelial species into two genera (i.e., *Borrelia* for RFG species, and *Borreliella* for LG species) [3,4], this proposal is still controversial; hence, herein we opted to consider all borrelial species as belonging to the genus *Borrelia*, as recently discussed [5,6].

Most RFG borreliae are primarily associated with soft ticks of the genus *Ornithodoros*, in which the bacteria perpetuate through transstadial and transovarial passages [7]. Given the great capacity of *Ornithodoros* ticks to survive for several years without feeding, these arthropods are also pointed out as main reservoirs of RFG borreliae [7]. Once transmitted to vertebrates upon tick feeding, RFG borreliae replicate in the blood of competent hosts, which might suffer clinical illness. In humans, this condition is reported as ‘tick-borne relapsing fever’ [2,7]. RFG borreliae are maintained in enzootic cycles, mostly between soft ticks (*Ornithodoros* spp.) and rodents [7,8]. Once infected, rodents develop an initial peak of spirochetemia that lasts for a few days, followed by new spirochete relapses interspaced by a few days during an infection course of usually one month, when signs of illness might or might not be present [9,10].

Among more than 20 species of RFG borreliae described in different continents of the world, most are pathogenic for humans and associated with *Ornithodoros* ticks [8]. Although tick-borne relapsing fever was first reported during the 19th century, it remains neglected as a human disease and little is known on its maintenance in ticks and vertebrates [7,8]. In South America, only two *Ornithodoros*-associated *Borrelia* species have been described, *Borrelia brasiliensis* and *Borrelia venezuelensis*; the latter was associated with clinical cases of human relapsing fever in Colombia and Venezuela during the first half of the 20th century [11].

The first report of RFG *Borrelia* associated to *Ornithodoros* ticks from Brazil was performed by Davis [12], who observed spirochetes in the blood of mice that were infested with *Ornithodoros brasiliensis* ticks from Rio Grande do Sul state. Although the spirochetes were named as *B. brasiliensis,* the isolate was lost and never reported again. During this century, Muñoz-Leal et al. [13] isolated *B. venezuelensis* by feeding *Ornithodoros rudis* from Maranhão state on Vesper mice (*Calomys callosus*). Subsequently, this isolate (designated as *B. venezuelensis* RMA01) was cultured in vitro and its genome sequenced [14]. *Borrelia venezuelensis* RMA01 constitutes to date the sole isolate of a RFG borreliae transmitted by an *Ornithodoros* tick in South America [11]. In a recent study, Muñoz-Leal et al. [15] reported by molecular methods four novel RFG *Borrelia* genotypes in human-biting *Ornithodoros* ticks from Brazil: *Borrelia* sp. Omi2MT and Omi3MT in *Ornithodoros mimon* from Mato Grosso state, *Borrelia* sp. JericoCE in *Ornithodoros hasei, Borrelia* sp. OrietCE in *Ornithodoros rietcorreai,* and *Borrelia* sp. TabajaraCE in *Ornithodoros tabajara*; the latter three tick species were collected in Ceará state, within the Caatinga semiarid biome of Brazil. Despite these findings, human infection by RFG borreliae in Brazil remains unreported [16].

Based on the recent advances on the occurrence of RFG borreliae associated to *Ornithodoros* ticks in Brazil, especially in the Caatinga biome, the present study aimed to isolate borrelial organisms from *Ornithodoros* ticks collected in additional areas of this ecosystem. For this purpose, collected *Ornithodoros* ticks were allowed to feed on guinea pigs, which were monitored for successful borrelial isolation, and subsequent molecular characterization.

## 2. Materials and Methods

### 2.1. Study Sites and Collection of Ornithodoros Ticks 

**Figure 1:**
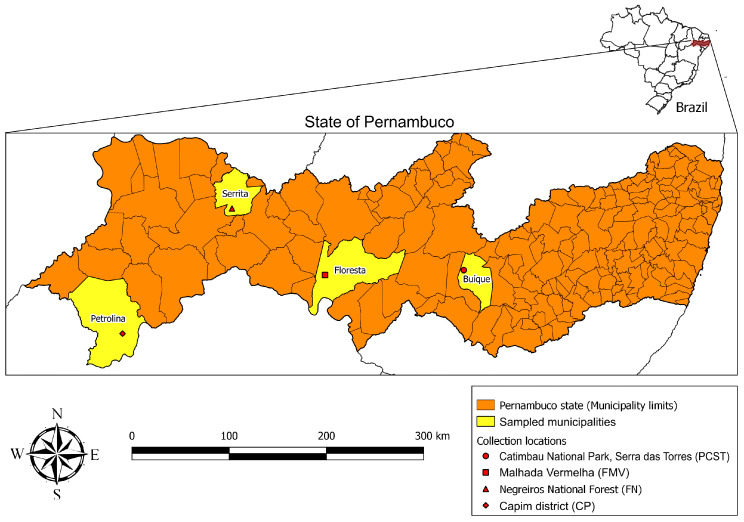
Locations in the state of Pernambuco, northeastern Brazil, where *Ornithodoros* ticks were collected.

During a field expedition in October 2019, ticks were collected from the environment in four localities in the state of Pernambuco, northeastern Brazil: (i) Catimbau National Park, Serra das Torres, Buíque municipality (08°34′0.3″ S, 37°14′27.8″ W; elevation 777 m); (ii) Malhada Vermelha, Floresta municipality (08°36′44.2″ S, 38°32′29.9″ W; 377 m); (iii) Negreiros National Forest, Serrita municipality (07°59′22.0″ S, 39°24′46.1″ W; 475 m); and (iv) Capim District, Petrolina municipality (09°09′40.3″ S, 40°26′16.2″ W; 454 m) (Figure 1). The four localities are located within the Caatinga biome, which is characterized by a semiarid climate (temperatures averaging 27 °C throughout the year, mean annual rainfall typically <500 mm), and deciduous vegetation composed typically of xeric shrub land and thorn forest that consist primarily of small, thorny trees that shed their leaves seasonally [17]. The prospected environments included rock formations with vestiges (e.g., feces) of wild rodents, including visualizations of the rock cavy *Kerodon rupestris* (Rodentia: Caviidae). *Ornithodoros* ticks were collected with tweezers from under the rocks and stored in punctured plastic vials, which were kept in an improvised environmental chamber (1.5 L-plastic bottle with a piece of humid cotton on the bottom) until their arrival at the laboratory, where ticks were placed in an incubator at 26 °C and 80% relative humidity. Under this condition, field-collected engorged females oviposited fertile eggs that resulted in hatched larvae.

### 2.2. Taxonomic Identification of Ornithodoros Ticks

Unfed larvae born in the laboratory were killed in hot water, clarified with 25% KOH, and mounted on slides using Hoyer’s medium to observe morphological characters by optical microscopy (Olympus BX40 optical microscope, Olympus Optical Co., Ltd., Tokyo, Japan). Living adults and nymphs were visualized and counted under a stereomicroscope (Zeiss Stemi SV 11, Zeiss, Münich, Germany). Species were determined according to taxonomic keys [18] and original descriptions [19,20].

Identification of the ticks was complemented by molecular analysis. For this purpose, two or three adult specimens of each species were individually submitted to DNA extraction by the guanidine isothiocyanate and phenol/chloroform technique [21]. A PCR protocol targeting a ≈460 bp fragment of the tick mitochondrial 16S rRNA gene was performed as described [22]. A second PCR protocol targeting a ≈270 bp fragment of the nuclear *Histone 3* (H3) gene was performed as described [23]. Amplicons of the expected size were prepared for sequencing using Big Dye Terminator Cycle Sequencing kit (Applied Biosystems, Foster City, CA, USA), and sequenced in an ABI automated sequencer (Applied Biosystems/Thermo Fisher Scientific, model ABI 3500 Genetic Analyzer, Foster City, CA, USA) with the same primers used for PCR. Obtained sequences were assembled, and primer-trimmed with Geneious R9 [24], and submitted to a BLAST analysis (www.ncbi.nlm.nih.gov/blast, accessed on 1 September 2022) to infer closest identities with congeneric ticks [25].

### 2.3. Isolation of Spirochetes from Ornithodoros Ticks

Attempts to isolate viable spirochetes were performed using field-collected ticks, which were separated in groups according to locations and species. Seven guinea pigs were infested, each one with ticks of one species from a single location. For this purpose, unengorged ticks were released inside a plastic feeding chamber (6 cm diameter) previously glued with a skin compatible-adhesive (Kamar Products, Zionsville, IN, USA) on the shaved dorsum of the guinea pig. Two hours after being released in the feeding chambers, engorged ticks were recovered and placed in the incubator for further studies. A drop of blood (≈2.5µL) was daily obtained from each of the seven guinea pigs by ear vein-puncture, expressed onto glass slides, and observed by dark-field microscopy to detect the presence of motile spirochetes. The mean number of spirochetes per field was calculated by counting the total number of motile spirochetes in 50 microscope fields at 200x magnification, dividing it by 50; results as decimal numbers were always rounded up. Experimental animals not presenting motile spirochetes during the first 21 days were considered negative and were not bled anymore. If a guinea pig showed motile spirochetes during the first 21 days, daily examinations were extended until 52 days after tick infestations.

Spirochetemic guinea pigs were anesthetized (xylazine 5 mg/kg + ketamine 35 mg/kg) and 2 mL of blood was collected by intracardiac puncture at the 18th day after tick infestation. Part of the blood was submitted to DNA extraction (see below) and the other part was intraperitoneally inoculated into five new experimental animals (three newborn guinea pigs, one mouse and one hamster, each one receiving ≈0.300 mL of blood) to perform the first passage of spirochetes into experimental animals in the laboratory. These inoculated animals were also evaluated daily through dark-field microscopy of blood samples, as described above. Two (newborn guinea pigs) of the five new rodents, when showing >10 spirochetes/field, were anesthetized (xylazine + ketamine) and euthanized by exsanguination via the intracardiac route. In this case, the collected blood was immediately put in heparin tubes, centrifuged, and the plasma was aliquoted into 2 mL-cryotubes, which were stored at −80 °C, and then at liquid nitrogen for cryopreservation of the isolated spirochetes. Rectal temperature of all animals was measured daily throughout the study with a digital clinical thermometer.

### 2.4. Molecular Analyses

DNA extraction of guinea pig blood samples was performed using the DNeasy Blood and Tissue Kit (Qiagen, Valencia, CA, USA), and tested by conventional PCR protocols for amplification of fragments of the borrelial genes 16S ribosomal RNA (*rrs*), flagellin (*flaB*), glycerophosphodiester phosphodiesterase (*glpQ*), and DNA gyrase subunit B (*gyrB*) (Table 1). In addition, we performed a multilocus sequencing typing (MLST) scheme according to Margos et al. [26] for amplification of the borrelial genes *clpA*, *clpX*, *pepX*, *pyrG*, *recG*, *rplB, nifS* and *uvrA* (Table S1—Supplementary Materials). DNA of *Borrelia anserina* strain PB [27] was used as positive control in all reactions. Obtained amplicons were visualized with UV light through 1.5% agarose gels stained with SYBR Safe (Thermo Fisher Scientifific, Waltham, MA, USA). Products containing a single expected size fragment were treated with ExoSAP-IT (USB Corporation, Cleveland, OH, USA) and prepared for sequencing with the BigDye kit (Applied Biosystems, Foster City, CA, USA). An ABI PRISM 3500 Genetic Analyzer (Applied Biosystems, Foster City, CA, USA) was employed for sequencing using the same primers to perform PCRs. Obtained sequences were assembled, trimmed, and translated to amino acid (if applicable) with Geneious R9 [24]. Generated DNA sequences were submitted to BLAST analysis (www.ncbi.nlm.nih.gov/blast, accessed on 1 November 2022) to infer closest identities with other spirochetes [25].

### 2.5. Phylogenetic Analyses

Obtained consensus sequences and orthologous sequences retrieved from GenBank were aligned with MAFFT using default parameters [31]. Phylogenetics trees were inferred using Maximum likelihood (ML) and Bayesian inference (BI) methods in IQ-TREE v 1.6.12 [32] and MrBayes v 3.2.6 [33], respectively. Protein-coding gene present distinct nucleotide exchange rates (heterogeneity) at the first, second, and third codon positions so datasets were partitioned into three codon positions (position-1, position-2, and position-3) [33,34]. For ML analyses, the best-fit models for non-coding and protein-coding genes datasets were calculated with the ModelFinder commands “TESTNEWONLY -mrate G” and “TESTNEWONLYMERGE -mrate G”, respectively [35]. Trees were run with rapid hill-climbing approach and stochastic disturbance applying 1,000 ultrafast bootstrapsing pseudo-replicates (UFB) to evaluate tree robustness. UFB values < 70%, between 70–94%, and ≥95% were considered non-significant, moderate, and high statistical support, respectively [36].

BI phylogenies were constructed implementing the MrBayes commands “lset nst = mixed rates = gamma” and “lset = mixed rates = invgamma” for non-coding and protein encoding datasets, respectively [33,37,38]. BI analyses were run with two independent tests of 20 × 10^6^ generations, each with four simultaneous Monte Carlo Markov chains (MCMC), sampling trees every 1000 generations, removing the first 25% as burn-in. Tracer software was used to confirm the MCMCs correlation as well as reached stationarity and effective sample size (ESS) [39]. All best-fit substitution models and partitions schemes were selected according to the Bayesian Information Criterion (BIC) [40]. Nodes with Bayesian posterior probabilities (BPP) values ≥ 0.70 were considered of high statistical support [41].

All trees were visualized and edited using FigTree v 1.4.1 (http://tree.bio.ed.ac.uk/software/figtree/, accessed on 1 September 2022) and Inkscape v 1.1 (https://inkscape.org/es/, accessed on 1 September 2022). Congruent topologies between ML and BI analyses were used to produce strict consensus trees in Geneious Prime with the Consensus Tree Builder tool, implementing a support threshold of 100% (www.geneious.com, accessed on 1 September 2022). The consensus phylogram included all monophyletic clades after comparing ML and BI topologies for each dataset.

### 2.6. Ethics Statement

Field collections of ticks were authorized by Instituto Chico Mendes de Conservação da Biodiversidade (ICMBio permit Sisbio 65137-1). Animal experimentation was approved by the Ethic Committee on Animal Use of the Faculty of Veterinary Medicine of the University of São Paulo (projects number 4425171018 and 2655061218).

## 3. Results

### 3.1. Collected Ornithodoros Ticks

A total of 1505 tick specimens were collected in four localities. Morphological analyses resulted in the identification of two *Ornithodoros* species: 932 specimens of *O. rietcorreai* from Buíque, Floresta, Serrita and Petrolina, and 573 specimens of *O. tabajara* from the former three localities. Microscopical analyses of laboratory-reared unfed larvae in mounted slides showed morphological characters corresponding to the species of their respective parental specimens.

Partial sequences of the mitochondrial 16S rRNA gene were generated for two *O. rietcorreai* specimens; one from Buíque, which was 99% (422/427 bp) identical to *O. rietcorreai* from Ceará state, Brazil (GenBank MT021433), and the other from Petrolina, which was 99% (425/426 bp) identical to *O. rietcorreai* from Paraíba state, Brazil (GenBank KX130781). The two *O. rietcorreai* haplotypes from this study were 96% identical to each other.

Partial sequences of the mitochondrial 16S rRNA gene were generated for three *O. tabajara* adult specimens, which were 92% (396/429 bp) identical to *O. tabajara* from Ceará state, Brazil (GenBank MT021434). The sequences of two specimens (one from Buíque and another from Floresta) were 100% identical to each other, whereas the third specimen (from Serrita) generated a second haplotype that differed by a single nucleotide polymorphism (99%; 428/429 bp) from the other two specimens. Although the external morphology of the ticks from Buíque, Floresta and Serrita was compatible with *O. tabajara,* we are provisionally treating them as *Ornithodoros* cf. *tabajara* due to the relatively high polymorphism (8% difference) of their 16S rDNA partial sequences with the type sequence of *O. tabajara* (MT021434) reported by Muñoz-Leal et al. (2021b). Partial sequences (216 bp) of the nuclear H3 gene of the three specimens of *O.* cf. *tabajara* were identical to each other, and by BLAST analysis, they were 99% (174/175 bp) identical to *O. tabajara* from Ceará (OK247605). Ongoing studies are in progress to elucidate the taxonomic status of the *O.* cf. *tabajara* ticks collected in the present study.

### 3.2. Isolation of Spirochetes from Ornithodoros Ticks

**Figure 2:**
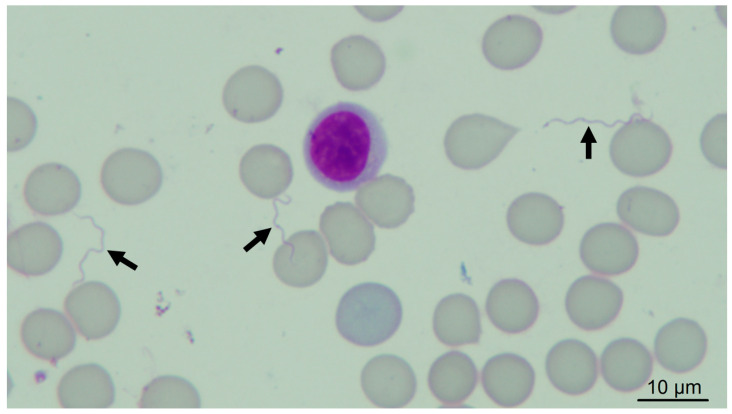
Giemsa-stained blood smear of guinea pig, showing spirochetes (arrows); original magnification: 1000×.

Four guinea pigs (numbers 1, 3, 5, 7) were infested with 925 *O. rietcorreai* ticks from four localities (25 to 445 ticks per guinea pig), and another three guinea pigs (numbers 2, 4, 6) were infested with 568 *O.* cf. *tabajara* ticks from three localities (136 to 289 ticks per guinea pig) (Table 2). Although we did not count the exact number of ticks that were fully engorged two hours after been released in the feeding chambers, nearly all of them became at least partially engorged. No spirochetes were visualized by dark-field microscopy in the blood of any of the four *O. rietcorreai-*infested guinea pigs (1, 3, 5, 7) during 21 consecutive days after infestation. Spirochetes were visualized in the three guinea pigs (2, 4, 6) that were infested with *O.* cf. *tabajara* ticks from three localities (Figure 2) (Video S1—Supplementary Materials).

**Figure 3:**
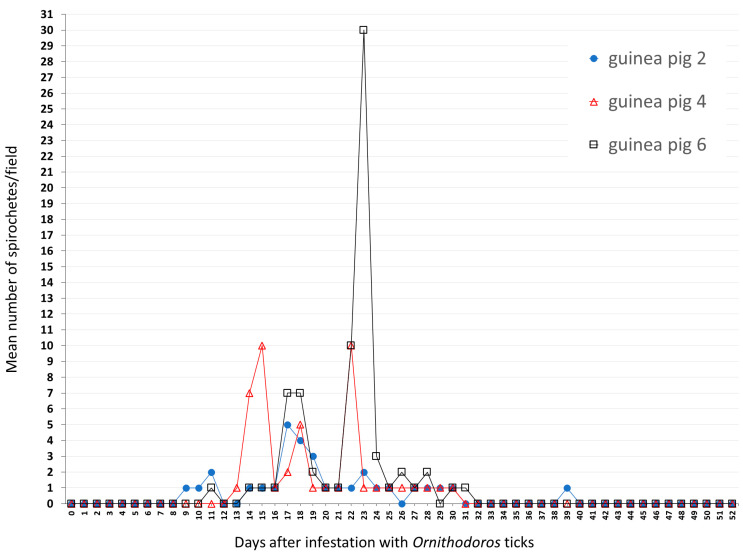
Results of dark-field examination of blood samples of guinea pigs according to the number of days after infestation with *Ornithodoros* cf. *tabajara* from Buíque (guinea pig 2), Floresta (guinea pig 4) and Serrita (guinea pig 6). Values presented as the mean number of motile spirochetes per microscope field at 200× magnification in each sampled day.

During the 52-day course of dark-field microscopy monitoring of guinea pig 2 (infested with *O.* cf. *tabajara* from Buíque), motile spirochetes were visualized in blood at 9 to 11, 14 to 25, 27 to 30, and 39 days after tick infestation; a mean of ≤1 spirochete/microscope field was visualized generally, although the maximum mean count was five spirochetes/field at day 17 (Figure 3). In guinea pig 4 (infested with *O.* cf. *tabajara* from Floresta), motile spirochetes were visualized in blood from 13 to 30 days after tick infestation; while a mean of ≤1 spirochete/field was visualized at most of the times, maximum mean values were 10 spirochetes/field at days 15 and 22 (Figure 3). In guinea pig 6 (infested with *O.* cf. *tabajara* from Serrita), motile spirochetes were visualized in blood at 11, 14 to 28, and 30 to 31 days after tick infestation; similarly, a mean of ≤1 spirochete/field was visualized at most of the times, yet a maximum mean value of 30 spirochetes/field was observed at day 23 (Figure 3). The three isolates of spirochetes were recovered from guinea pigs numbers 2, 4, and 6 infested with *O.* cf. *tabajara* ticks, and were named Buíque-PCST, Floresta-FMV, and Serrita-FN, respectively.

**Figure 4:**
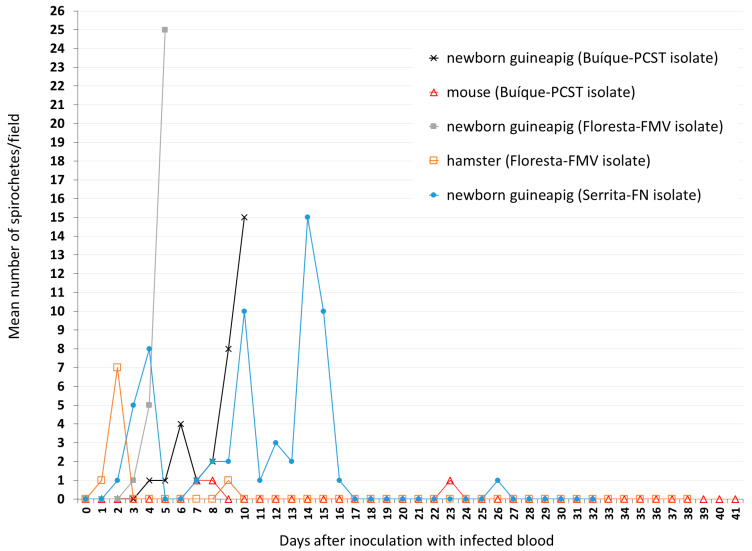
Results of dark-field examination of blood samples of newborn guinea pigs, hamster, and mouse according to the number of days after inoculation with blood samples that were collected from guinea pigs showing spirochetemia due to *Borrelia* sp. isolate Buíque-PCST or Floresta-FMV or Serrita-FN. Values presented as the mean number of motile spirochetes per microscope field at 200x magnification in each sampled day.

Guinea pigs 2, 4, and 6 were bled at the 18th day after tick infestation (when the mean numbers of spirochetes/field were 4, 5, and 7, respectively), and their blood samples were inoculated into other animals to perform the first experimental animal passage of the spirochetes. In this case, the blood of guinea pig 2 (isolate Buíque-PCST) was inoculated into a newborn guinea pig and a mouse (Table 2). Blood samples of this newborn guinea pig showed motile spirochetes (mean: ≤1 to 15 spirochetes/field) from the 4th to the 10th day after inoculation (Figure 4), when it was euthanized by exsanguination and its plasma cryopreserved. Dark-field microscopy of the inoculated mouse revealed spirochetes only at the 7th, 8th, and 23th days after inoculation (mean ≤1 spirochete/field), despite of this experimental animal being daily examined until the 41st day (Figure 4). The blood of guinea pig 4 (isolate Floresta-FMV) was inoculated into a newborn guinea pig and a hamster (Table 2). Blood samples of this newborn guinea pig showed motile spirochetes (mean: ≤1 to 25 spirochetes/field) from the 3rd to the 5th day after inoculation (Figure 4), when it was euthanized by exsanguination and its plasma cryopreserved. Dark-field microscopy of the inoculated hamster revealed spirochetes only at the 1st, 2nd, and 9th days after inoculation (mean ≤1 or 7 spirochetes/field), despite of this experimental animal being daily examined until the 38th day (Figure 4). Finally, the blood of guinea pig 6 (isolate Serrita-FN) was inoculated into a newborn guinea pig, which showed motile spirochetes in blood at 2 to 4, 7 to 16, and 26 days after inoculation, with mean numbers of spirochetes/field varying from ≤1 to 15 (Figure 4). No experimental animal developed fever or clinical abnormalities during the present study.

### 3.3. Molecular Characterization of Spirochetes

PCR assays resulted in the successful amplification of fragments of four borrelial genes (*rrs*, *flaB*, *glpQ*, *gyrB*) from blood samples that were collected from spirochetemic guinea pigs 2, 4, and 6. For each borrelial gene, sequences were identical among the three guinea pigs, indicating that isolates Buíque-PCST, Floresta-FMV, and Serrita-FN represented the same *Borrelia* species. Results of BLAST analyses with 100% query cover showed that a 1410 bp-fragment of the *rrs* gene was >99.2% identical to the sequences of *Borrelia hispanica* (DQ057988, GU350705), *Borrelia duttonii* (CP000976, GU350711), and *Borrelia crocidurae* (CP003426, DQ057990); a 614 bp-fragment of the *flaB* gene was most identical (99.3%) to *Borrelia* sp. clone TabajaraCE from *O. tabajara,* Brazil (MT076263); a 450 bp-fragment of the *glpQ* gene was most identical (89.3%) to *B. crocidurae* (CP003426); and a 417 bp-fragment of the *gyrB* gene was most identical (89.9%) to *B. crocidurae* (CP004267) and *B. duttonii* (CP000976).

**Figure 5:**
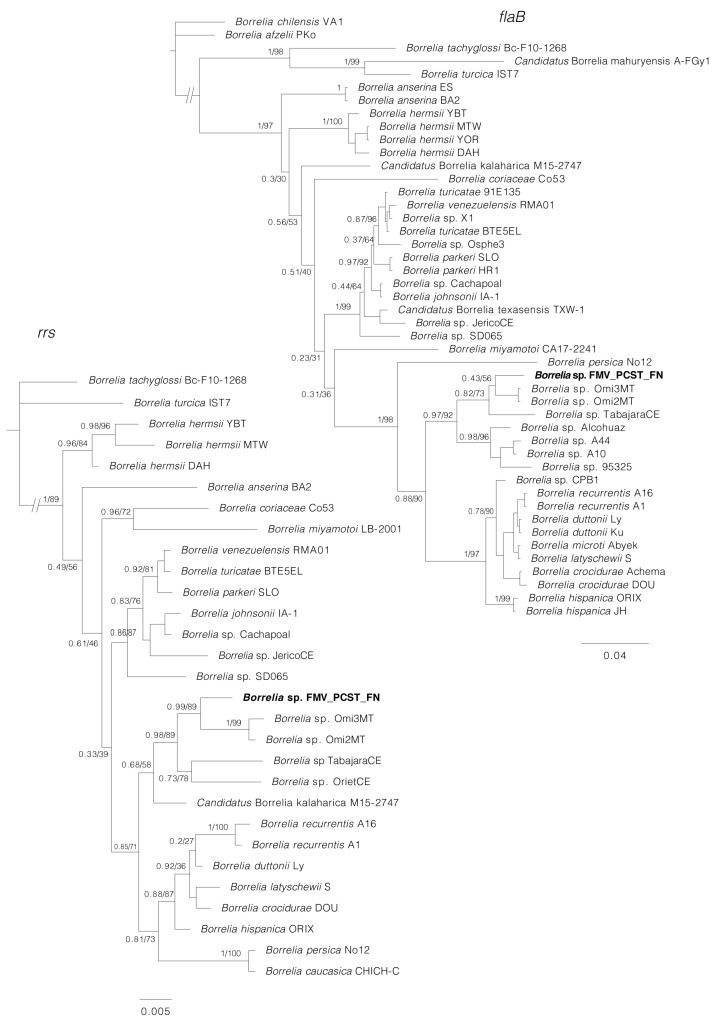
Phylogenetic analyses of relapsing fever group (RFG) *Borrelia* spp. inferred for *rrs* and *flaB* partial sequences. The *rrs* tree is based on 29 sequences and an alignment of 1343 base pairs; best-fit evolutionary models calculated for ML and BI methods were TPM3u + F + G4; and *M*_90_, *M*_15_, *M*_183_, *M*_177_, *M*_152_, *M*_85_, respectively. The *flaB* tree is based on 46 sequences and an alignment of 638 base pairs; best-fit evolutionary models calculated for ML and BI methods were TVM + F + G4 (position-1), TPM2u + F + G4 (position-2), HKY + F + G4 (position-3); and *M*_95_, *M*_27_ (position-1); *M*_34_, *M*_123_, *M*_129_, *M*_127_ (position-2); *M*_50_, *M*_152_, *M*_15_, *M*_90_, *M*_147_, *M*_157_ (position-3), respectively. The sequence in bold (*Borrelia* sp. FMV_PCST_FN) represents the consensus of isolates Buíque-PCST, Floresta-FMV, and Serrita-FN from this study. Trees are drawn to scale. Numbers above or below tree branches represent Bayesian posterior probabilities/ML bootstrap values. Scale bar indicates nucleotide substitutions per site. GenBank accession numbers of the public sequences used for *Borrelia* phylogenies reconstruction based on *rrs* and *flaB* genes are shown in Table S2.

**Figure 6:**
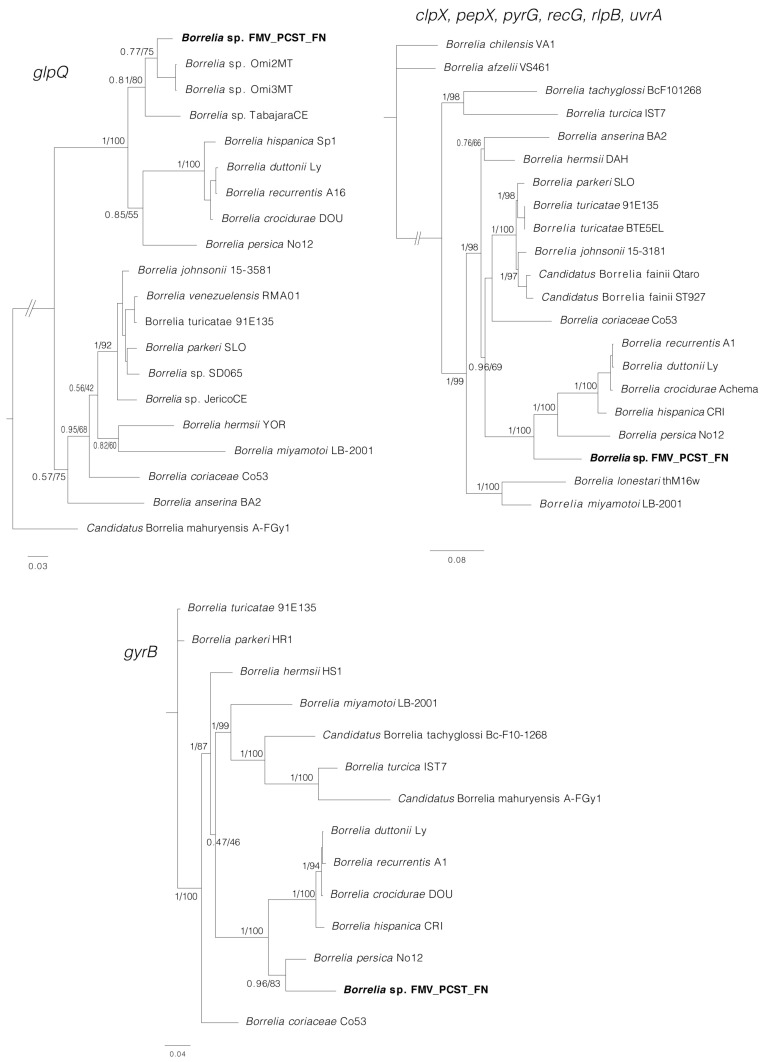
Phylogenetic analyses of relapsing fever group (RFG) *Borrelia* spp. inferred for *glpQ, gyrB*, and concatenated *clpX, pepX, pyrG, recG, rlpB, uvrA* (MLST). The *glpQ* tree is based on 20 sequences and an alignment of 535 base pairs; best-fit evolutionary models calculated for ML and BI methods were TIM + F+I + G4 (position-1 and position-2), K3Pu + F+I + G4 (position-3); and *M*_134_, *M*_200_, *M*_189_, *M*_198_, *M*_166_, *M*_203_(position-1, 2, and 3) respectively. The *gyrB* tree is based on 14 sequences and an alignment of 1917 base pairs; best-fit evolutionary models calculated for ML and BI methods were K3Pu + F+I + G4 (position-1), TIM3 + F+I + G4 (position-2), TVM + F+I + G4 (position-3); and *M*_125_, *M*_50_, *M*_189_, *M*_193_, *M*_157_, *M*_147_ (position-1); *M*_125_, *M*_134_, *M*_60_, *M*_189_ (position-2); *M*_147_, *M*_195_, *M*_189_, *M*_157_, *M*_203_ (position-3), respectively. The MLST tree is based on 21 sequences and an alignment of 4,788 base pairs; best-fit evolutionary models calculated for ML and BI methods were GTR + F+G4 (position-1), GTR + F+G4 (position-2), GTR + F+I + G4 (position-3); and *M*_202_, *M*_175_, *M*_193_, *M*_203_ (position-1); *M*_200_, *M*_203_, *M*_198_, *M*_134_, *M*_190_, *M*_189_, *M*_160_ (position-2); *M*_195_, *M*_157_, *M*_147_ (position-3), respectively. The sequence in bold (*Borrelia* sp. FMV_PCST_FN) represents the consensus of isolates Buíque-PCST, Floresta-FMV, and Serrita-FN from this study. Trees are drawn to scale. Numbers above or below tree branches represent Bayesian posterior probabilities/ML bootstrap values. Scale bar indicates nucleotide substitutions per site. GenBank accession numbers of the public sequences used for *Borrelia* phylogenies reconstruction based on *glpQ* and *gyrB* genes are shown in Table S2. Sequence Type (ST) numbers of the sequences used for *Borrelia* phylogenies reconstruction based on MLST genes are shown in Table S3.

Phylogenetic analyses inferred from partial sequences of each of four genes (*rrs*, *flaB*, *glpQ*, *gyrB*) showed that in all cases, the sequences generated for *Borrelia* sp. (isolates Buíque-PCST, Floresta-FMV, and Serrita-FN) grouped within a clade composed by *B. recurrentis* and tick-borne relapsing fever borreliae of the Old World, such as *B. hispanica*, *B. duttonii*, *B. crocidurae*, and *Borrelia persica* (Figure 5 and Figure 6). For the borrelial genes *rrs, flaB* and *glpQ,* this clade also included genotypes of unnamed *Borrelia* spp. recently reported in *Ornithodoros* ticks from Brazil [15], such as *Borrelia* sp. Omi2MT and *Borrelia* sp. Omi3MT from *O. mimon*, *Borrelia* sp. TabajaraCE from *O. tabajara*, and *Borrelia* sp. OrietCE from *O. rietcorreai* (this latter one only for the *rrs* gene). Finally, in the *rrs, flaB* and *glpQ* phylogenetic trees, this large clade was sister to another large clade that contained *Borrelia* species associated to *Ornithodoros* ticks from the New World (such as the North American agents *Borrelia turicatae, Borrelia parkeri* and *Borrelia johnsonii*), and two agents from Brazil (*B. venezuelensis* from *O. rudis,* and *Borrelia* sp. JericoCE from *O. hasei*).

PCR amplification and DNA sequences were obtained for six of the eight MLST loci (*clpX*, *pepX*, *pyrG*, *recG*, *rplB,* and *uvrA*) from guinea pig blood samples. Pairwise comparisons proved that the *Borrelia* sequences from guinea pigs 2, 4, and 6 were identical with each other. The phylogenetic analysis of concatenated MLST sequences (Figure 6) corroborates the previous trees, indicating that *Borrelia* sp. (isolates Buíque-PCST, Floresta-FMV, and Serrita-FN) belongs to the RFG borreliae, in which it grouped within a clade that included *B. recurrentis* and tick-borne relapsing fever borreliae of the Old World (*B. hispanica*, *B. duttonii*, *B. crocidurae* and *B. persica*). This clade was sister to a large clade composed mostly by North American agents (*B. turicatae, B. parkeri* and *B. johnsonii*) associated with *Ornithodoros* spp.

## 4. Discussion

In this study we obtained three primary isolates (Buíque-PCST, Floresta-FMV, and Serrita-FN) of a novel RFG *Borrelia* species through the feeding of *O.* cf. *tabajara* ticks upon guinea pigs, which showed spirochetemia between 9 and 39 days after tick feeding. Guinea pig infection was confirmed by passage into experimental animal, based on the inoculation of guinea pig infected blood in newborn guinea pigs, mouse, and hamster. Although the three borrelial isolates were from three geographically distinct populations of *O.* cf. *tabajara* ticks, molecular analyses indicated that the three populations were infected by the same borrelial organism, as they showed identical DNA partial sequences of 10 *Borrelia* genes, *rrs*, *flaB*, *glpQ*, *gyrB*, *clpX*, *pepX*, *pyrG*, *recG*, *rplB,* and *uvrA.* Phylogenetic analyses based on these partial sequences indicated that isolates Buíque-PCST, Floresta-FMV, and Serrita-FN represent a distinct taxon that is more closely related to Old World *Ornithodoros-*associated *Borrelia* species than to New World borreliae (including *B. venezuelensis,* which was recently isolated from *O. rudis* from Brazil [13]).

In the phylogenetic analyses inferred from partial sequences of the borrelial genes *rrs*, *flaB* and/or *glpQ*, the isolates Buíque-PCST, Floresta-FMV, and Serrita-FN formed a monophyletic group with borrelial agents recently reported by molecular methods in *Ornithodoros* ticks from Brazil [15]. However, the phylogenetic distances between the herein characterized agent and these previous borrelial haplotypes were higher than the distances between several RFG *Borrelia* valid species (Figure 5 and Figure 6), reinforcing that isolates Buíque-PCST, Floresta-FMV and Serrita-FN represent a new RFG taxon. Interestingly, the insertion of this Brazilian monophyletic group within a large clade composed by *Ornithodoros*-associated RFG borreliae from the Old World (*B. hispanica*, *B. duttonii*, *B. crocidurae*, and *B. persica*) refute the classical paradigm that *Ornithodoros*-associated RFG borreliae are divided into two clades, one composed by Old World species, and another by New World species [8]. Undoubtedly, the historical support of this hypothesis was related to the little exploration of the diversity of RFG borreliae in many parts of the world, especially in South America. The improvement in phylogenetic tools adopted in recent studies have pointed out this division as rather artificial [7]. For instance, at least two novel RFG borrelial agents from Africa (an unnamed *Borrelia* sp. and ‘*Candidatus* Borrelia fainii’) were shown by phylogenetic analyses to belong to the classical New World clade of RFG *Borrelia* spp. [42,43].

The procedure of feeding field-collected ticks on laboratory animals (i.e., xenodiagnosis) has been used to recover spirochetes before cultivation in axenic media [44]. When this procedure is successful, it also demonstrates vector competence. Hence, herein we demonstrated that *O.* cf. *tabajara* is a competent vector of *Borrelia* sp. isolates Buíque-PCST, Floresta-FMV, and Serrita-FN. Previous studies demonstrated that the characterization of RFG borreliae through experimental infection of rodents resulted in variable degrees of clinical signs and borrelial pathogenicity [8]. In the present study, none of the infected rodents developed fever or any clinical sign of illness during the evaluated period, including during the highest peaks of spirochetemia. This finding is similar to studies with *B. hermsii,* which induced no signs of illness in chipmunks (*Eutamias amoenus*) and meadow voles (*Microtus pennsylvanicus*) during spirochetemia [9], and with *Borrelia crocidurae*, which also did not induce clinical signs in the multimammate rat during spirochetemia (*Mastomys natalensis*) [10]. In contrast, *B. hermsii* induced clinical illness coincidently with spirochetemia in pine squirrels (*Tamiasciurus hudsonicus richardsoni*) [9]. Since there is no correlation between the pathogenicity of a borrelial agent to humans and to laboratory animals, it is not known if *Borrelia* sp. isolates Buíque-PCST, Floresta-FMV, and Serrita-FN are capable of causing relapsing fever in humans.

Even though we used only one laboratory mouse and one hamster for experimental infections, guinea pigs were clearly more susceptible than the former two rodents to the infection by *Borrelia* sp. isolates Buíque-PCST, Floresta-FMV, and Serrita-FN; i.e., during spirochetemia peaks, mean number of spirochetes/microscopy field varied from 10 to 30 in all but one guinea pigs, contrasting to maximal peaks of ≤1 in a mouse and 7 spirochetes/microscopy field in a hamster (Figure 3 and Figure 4). Spirochetemia in guinea pigs were observed up to ≈30 days, similarly to previous studies using different rodent species infected with *B. hermsii* or *B. crocidurae* [9,10,45]. The spirochetemic period in guinea pigs was characterized by two or three peaks interspersed by few days with no or very low spirochetemia (≤1 spirochete/microscopy field). This pattern has been reported for RFG borreliae and is related to antigenic variation of bacterial major surface immunogenic proteins [variable major proteins (Vmps)] during the infection period [8].

Most of the RFG borreliae are maintained in enzootic cycles between *Ornithodoros* ticks and rodents [7,8]. The two tick species of the present study, *O. rietcorreai* and *O.* cf. *tabajara*, were collected from rocky formations inhabited by rodents, including the Caviidae rock cavy. In fact, this rodent species is regarded as one of main hosts for *O. rietcorreai* [19] and possibly also for *O. tabajara* [20]. This condition motivated us to test a laboratory Caviidae species (guinea pig, *Cavia porcellus*) for isolation of borreliae, given the phylogenetic close relatedness between rocky cavy and guinea pig [46]. Indeed, our results of spirochetemic guinea pigs claim for additional field studies to explore the occurrence of natural borrelial infection in rocky cavy and other Caviidae species living in rocky formations with the presence of *O.* cf. *tabajara,* and the likely participation of these rodents in the enzootic cycle of *Borrelia* sp. isolates Buíque-PCST, Floresta-FMV, and Serrita-FN.

Although *O.* cf. *tabajara* was collected in sympatry with *O. rietcorreai* in the prospected environments of the present study, borreliae were not observed in the guinea pigs infested with the latter tick species. This condition suggests a specificity affinity of isolates Buíque-PCST, Floresta-FMV, and Serrita-FN to *O.* cf. *tabajara.* In fact, a strict host specificity between borreliae and *Ornithodoros* ticks has been reported for most of the RFG agents [7,8]. On the other hand, we cannot exclude the possibility that *O. rietcorreai* specimens were infected by any of the current isolates, since we did not evaluate ticks by direct methods such as PCR targeting borrelial genes. In addition, it is also possible that those *O. rietcorreai* specimens were carrying another borrelial agent not infective for guinea pigs. For instance, Muñoz-Leal et al. [15] reported molecular detection of another RFG agent (*Borrelia* sp. OrietCE) in *O. rietcorreai* from Ceará state (Caatinga biome), suggesting that this tick species might also be associated with a specific borrelial agent.

Although the *O.* cf. *tabajara* ticks presented morphological features compatible with the original description of *O. tabajara* from Ceará state, their 16S rDNA sequences were 8% different, indicating that they could represent different species or two different lineages of *O. tabajara.* Interestingly, the original *O. tabajara* ticks from Ceará were found infected by another borrelial agent, *Borrelia* sp. TabajaraCE [15]. In our phylogenetic analyses inferred from partial sequences of the *rrs*, *flaB* and *glpQ* genes (Figure 5 and Figure 6), *Borrelia* sp. TabajaraCE was shown to be distinct but closely related to the *O.* cf. *tabajara* isolates (Buíque-PCST, Floresta-FMV, and Serrita-FN). Indeed, these findings are coherent with the paradigm of strict host specificity between borreliae and *Ornithodoros* ticks [7,8].

Based on the unique genetic profile of isolates Buíque-PCST, Floresta-FMV, and Serrita-FN, we propose to name them as ‘*Candidatus* Borrelia caatinga’, in allusion to their geographical origin, the Brazilian Caatinga biome. However, we are aware that a formal description and validation of the taxon ‘*Ca.* B. caatinga’ needs to be performed in a near future after its establishment in axenic media and determination of its entire genome.

## Figures and Tables

**Table 1:** List of the primer pairs used in the study for amplification of four *Borrelia* genes by conventional PCR assays.

Genes and Primers	DNA Sequences (5′-3′) of Forward (F) and Reverse (R) Primers of Each Pair of Primers	Amplicon Size (bp)	References
*rrs*			
FD3	F- AGAGTTTGATCCTGGCTTAG	1540	[28]
T50	R- GTTACGACTTCACCCTCCT		
FD3	F- AGAGTTTGATCCTGGCTTAG	729 *	[29]
16S-1	R- TAGAAGTTCGCCTTCGCCTCTG		
16S-2	F- TACAGGTGCTGCATGGTTGTCG	513 *	[29]
T50	R- GTTACGACTTCACCCTCCT		
Rec-4	F- ATGCTAGAAACTGCATGA	520 *	[28]
Rec-9	R- TCGTCTGAGTCCCCATCT		
*flaB*			
FlaLL	F- ACATATTCAGATGCAGACAGAGGT	665	[30]
FlaRL	R- GCAATCATAGCCATTGCAGATTGT		
FlaLL	F- ACATATTCAGATGCAGACAGAGGT	485 *	[30]
FlaRS	R- CTTTGATCACTTATCATTCTAATAGC		
FlaLS	F- AACAGCTGAAGAGCTTGGAATG	522 *	[30]
FlaRL	R- GCAATCATAGCCATTGCAGATTGT		
*glpQ*			
glpQ F + 1	F- GGGGTTCTGTTACTGCTAGTGCCATTAC	1386	[29]
Rev-2	R- CAATACTAAGACCAGTTGCTCCTCCGCC		
glpQ F + 1	F- GGGGTTCTGTTACTGCTAGTGCCATTAC	802 *	[29]
glpQ F − 1	R- CAATTTTAGATATGTCTTTACCTTGTTGTTTATGCC		
*gyrB*			
gyrB 5′	F- GGTTTATGAGTTATGTTGCTAGTAATATTCAAGTGC	2026	[29]
gyrB 3′	R- GGCTCTTGAAACAATAACAGACATCGC		
gyrB 3′	F- GGTTTATGAGTTATGTTGCTAGTAATATTCAAGTGC	542 *	[29]
gyrB 5′ + 3	R- GCTGATGCTGATGTTGATGG		

* Amplified through a nested or heminested reaction.

**Table 2:** Results of infestation trials for isolation of spirochetes from *Ornithodoros* ticks that were collected in four localities of the state of Pernambuco, Caatinga biome, Brazil, during 2019.

Locality	Tick Species	No. Ticks Released in the Feeding Chamber of Guinea Pigs *^a^*	Isolation of Spirochetes though Guinea Pigs and Experimental Animals *^b^*			
			Guinea Pigs		Passages into Experimental Animals *^c^*	
			No.	Spirochetemia	Rodent	Spirochetemia
Buíque	*Ornithodoros rietcorreai*	25 (4F, 3M, 18N)	1	No		
	*Ornithodoros* cf. *tabajara*	143 (32F, 31M, 80N)	2	Yes	Guinea pig	Yes
					Mouse	Yes
Floresta	*O. rietcorreai*	365 (18F, 29M, 318N)	3	No		
	*O.* cf. *tabajara*	289 (44F, 51M, 194N)	4	Yes	Guinea pig	Yes
					Hamster	Yes
Serrita	*O. rietcorreai*	445 (16F, 34M, 395N)	5	No		
	*O.* cf. *tabajara*	136 (18F, 11M, 107N)	6	Yes	Guinea pig	Yes
Petrolina	*O. rietcorreai*	90 (3F, 4M, 83N)	7	No		

*^a^* F: females; M: males; N: nymphs; *^b^* A total of seven guinea pigs were used, each one infested ticks of one species from each locality. Infested guinea pigs were daily checked for spirochetemia by dark-field microscopy of blood samples from the first to at least the 21st day after tick infestation. *^c^* At the 18th day after tick infestation, spirochetemic guinea pigs (nos. 2, 4, 6) were bled and their blood was inoculated into experimental animals including new guinea pigs, mouse, and hamster, which were daily checked for spirochetemia by dark-field microscopy of blood samples.

## Data Availability

Partial sequences of ticks and borrelial genes generated in this study are available in GenBank under the accession numbers OP940118-OP940122 and OP937326-OP937328 for tick 16SrRNA and H3 genes, respectively; OP941213 for borrelial *rrs* gene, and OP952107-OP952115 for borrelial *flaB, glpQ, gyrB, clpX, pepX, pyrG, recG, rplB*, and *uvrA* protein genes. GenBank accession numbers of the public sequences used for *Borrelia* phylogenies reconstruction based on *rrs, glpQ*, *gyrB* and *flaB* genes are shown in Table S2. ST numbers of the sequences used for *Borrelia* phylogenies reconstruction based on MLST genes are shown in Table S3.

## Supplementary Materials

The following supporting information can be downloaded at: https://www.mdpi.com/article/10.3390/microorganisms11020370/s1, Table S1: List of primers used in the multilocus sequencing typing (MLST) scheme according to Margos et al. [26] for amplification of eight genes of *Borrelia* spp. of the relapsing fever group; Table S2: GenBank accession numbers of the public sequences used for *Borrelia* phylogenies reconstruction based on *rrs*, *flaB*, *glpQ* and *gyrB* genes; Table S3: Sequence Type (ST) numbers of the sequences used for *Borrelia* phylogenies reconstruction based on MLST genes; Video S1: Motile spirochetes in the blood of guinea pig 6, observed by dark-field microscopy (original magnification: 200×).
