# Supplementary material for: A Novel Relapsing Fever Group Borrelia Isolated from Ornithodoros Ticks of the Brazilian Caatinga
Source: Microorganisms. 2023 Feb 1;11(2):370. doi: 10.3390/microorganisms11020370 (PMC9964043; doi:10.3390/microorganisms11020370)
Supplement: Supplementary file 1 [file microorganisms-11-00370-s001.zip › Table S1 primers.pdf]

Table S1. List of primers used in the multilocus sequencing typing (MLST) scheme according to Margos et al. [29] for amplification of eight genes of *Borrelia* spp. of the relapsing fever group.

Gene	Primers	**DNA sequences (5'-3') of forward (F) and reverse (R) primers of each pair of primers**	**Amplicon size (bp)**	**Reference**
clpA	clpAF1262	F- AAGCTTTTGA**YY**TATTAGATGGTC	1014	http://pubmlst.org/borrelia
	clpAR2276	R- TCATATTT**D**AT**R**GT**D**TCGTC		
	clpAF1264	F- GCTTTTGAYYTATTAGATGGTC	834*	
	clpAR2098	R- CAAAAAAAA**S**ATC**H**A**RR**TTTTCATTTTTTAG		
rplB	rplF3	F- GGAGAAAAATATGGG**K**ATTAAGAC	766	http://pubmlst.org/borrelia
	rplR769	R- G**R**CCCCAAGG**W**GATAC		
	rplF18	F- ATTAAGACTTATA**R**GCCAAAAAC	741*	
	rplF759	R- GATACAGGATG**W**CGACC		
	rplF24	F- GACTTATA**R**GCCAAAAACTTC	741*	
	rplF765	R- CCAAGG**W**GATACAGGATG		
pyrG	pyrF372	F- TGG**R**AA**R**TATTTAGG**K**AGAACTG	1003	http://pubmlst.org/borrelia
	pyrR1375	R- TATTTAGG**K**AGAACTGTACAGC		
	PyrF379	F- TATTTAGG**K**AGAACTGTACAGC	873*	
	PyrR1252	R- TAT**R**CCACAAACATTACG**K**GC		
recG	recF898	F- GC**K**TTTCT**M**TCTAG**Y**ATTCC	881	http://pubmlst.org/borrelia
	recR1779	R- TC**R**GTTAAAGGTTCCTTATAAAG		
	recF908	F- CTAG**Y**ATTCCT**Y**TAATTGA**R**GC	871*	
	recR1779	R- TC**R**GTTAAAGGTTCCTTATAAAG		
clpX	clpXF109	F- GC**Y**ATTTGTTTTGAATGTTCTAAAATATG	859	http://pubmlst.org/borrelia
	clpXR1277	R- TAAAGTTCTTTTGCCCAAGG		
	clpXF268	F- GC**Y**ATTTGTTTTGAATGTTCTAAAATATG	721*	
	clpXR1183	R- CTTTTTTAATTTGCTTA**STW**GAAGG		
pepX	pepXF361	F- AGAGA**Y**TTAAG**Y**TTA**K**CAGG	1168	http://pubmlst.org/borrelia
	pepXR1207	R- C**Y**ATAGTTTCTCTTAAAGA**Y**TGC		
	pepXF364	F- GA**Y**TTAAG**Y**TTA**K**CAGGA**R**TTG	823*	
	pepXR1187	R- TGCATTCCCCACATTGG		
nifS	nifF23	F- TAAAAATAAAGAGTACTAAATAAATG	876	http://pubmlst.org/borrelia
	nifR899	R- CCAAGACCAATAATTCCTGC		
	nifF60	F- AAATAATGGTATAATTCTTAAAATAA	656*	
	nifR716	R- GGAGCAAGCATTTT**Y**TGTC		
uvrA	uvrF1170	F- GAGGCGTTATCTT**W**CAAC	983	http://pubmlst.org/borrelia
	uvrR2153	R- CTAATCTC**D**GTAAAAAATCCAACATAAG		
	uvrF1173	F- GCGTTATCTT**W**CAACTGAATC	974*	
	uvrR2147	R- TC**D**GTAAAAAATCCAACATAAGTTGC		

* amplified through a nested or heminested reaction.
